# Extracorporeal CO_2_ elimination for acute exacerbation of severe COPD requiring invasive mechanical ventilation: a randomized controlled trial (the X-COPD trial)

**DOI:** 10.1186/s13054-026-06300-6

**Published:** 2026-09-03

**Authors:** Christian Karagiannidis, Jordi Riera, Pablo Blanco-Schweizer, Stephan Strassmann, Michaela Merten, Daniel Brodie, Thomas Staudinger, Wolfram Windisch, Laurent Brochard, Arthur S. Slutsky

**Affiliations:** 1 ARDS and ECMO Centre Cologne-Merheim, University Witten/Herdecke, Ostmerheimer Str.200, 51109 Cologne, Germany; 2 Critical Care Department, Vall d’Hebron University Hospital, SODIR, Vall d’Hebron Research Institute, Barcelona, Spain; 3 Intensive Care Unit, Rio Hortega University Hospital, Valladolid, Spain; 4 Division of Pulmonary and Critical Care Medicine, Department of Medicine, Johns Hopkins University School of Medicine, Baltimore, MD USA; 5 Department of Medicine I, Medical University of Vienna, Intensive Care Unit 13i2, Währinger Gürtel 18–20, Vienna, 1090 Austria; 6 Interdepartmental Division of Critical Care Medicine, University of Toronto, Toronto, ON Canada; 7 Keenan Research Centre, Li Ka Shing Knowledge Institute, St Michael’s Hospital, Unity Health, Toronto, ON Canada

**Keywords:** COPD, Exacerbation, ECMO, Ventilation, NIV

## Abstract

**Rationale:**

Acute exacerbations of chronic obstructive pulmonary disease (AE-COPD) requiring invasive mechanical ventilation (IMV) are associated with high mortality and long-term disability. Extracorporeal CO₂ removal (ECCO₂R) using modern high-capacity devices may facilitate early endotracheal extubation and reduce IMV-related complications.

**Objectives:**

To evaluate whether ECCO_2_R-facilitated early extubation improves clinical outcomes compared with standard IMV in patients with severe AE-COPD requiring IMV.

**Methods:**

Adults with acute hypercapnic respiratory failure due to AE-COPD requiring IMV who failed or were ineligible for extubation within 24 hours of intubation were randomized to ECCO_2_R or no ECCO_2_R. The primary endpoint was a composite of death or severe disability at day 60.

**Results:**

18 patients were randomized before the trial was terminated early by the sponsor for financial reasons (planned enrollment: 192). The primary composite endpoint occurred in 0/8 ECCO_2_R-treated patients versus 3/9 evaluable IMV-treated patients (33%; risk difference −33%; 95% CI −65% to 6%; *p* = 0.21). IMV duration was shorter in the ECCO_2_R group (7.1 ± 2.0 vs. 24.3 ± 21.4 days; median 7.0 vs. 16.0 days; mean difference −17.2 days; *p* = 0.043), yielding, in a post hoc exploratory analysis, more overall device-free days at day 29 (17 ± 4 device-support-free days compared with 8 ± 7 days in the IMV group (*p* = 0.011)). Ventilator-associated pneumonia occurred in 0 versus 3 patients (37.5%), respectively. Sedation was discontinued earlier in the ECCO₂R group. Severe bleeding occurred in one ECCO₂R-treated patient (12.5%).

**Conclusions:**

In this prematurely terminated trial, ECCO₂R-facilitated early extubation using a device with a capacity to eliminate more than 50% of the average CO_2_ production, was associated with a shorter duration of invasive mechanical ventilation. Numerical trends favored ECCO_2_R across several secondary outcomes, although interpretation is limited by premature termination and the very small sample size. Adequately powered multicenter trials are warranted.

**Supplementary Information:**

The online version contains supplementary material available at https://doi.org/10.1186/s13054-026-06300-6.

## Introduction

Chronic obstructive pulmonary disease (COPD) is a leading cause of death worldwide, despite recent advances in therapy. Acute exacerbations, especially in patients treated with non-invasive (NIV) or invasive mechanical ventilation (IMV) are a major driver of severe disability or death. In-hospital mortality ranges between 10 and 15% for patients treated with NIV, and up to 40% in patients treated with IMV [1, 2]. Randomized controlled trials have demonstrated a remarkable advantage of avoiding invasive mechanical ventilation in favor of NIV, in particular by reducing infectious complications [3, 4]. A subset of these patients may require long-term IMV and are often difficult to wean from mechanical ventilation [5].

Veno-venous extracorporeal carbon dioxide (CO_2_) elimination (ECCO_2_R) is a technique aimed at removing CO_2_ and can be effective even at relatively low blood flow rates, ranging between 500 and 1500 ml/min [6–9], thus requiring smaller cannulas than are needed for full ECMO support at higher blood flow rates. Approximately 25% of an adult’s resting CO_2_ production can be removed at blood flow rates of 500 ml/min, and about 50% at a rate of 1000 ml/min, depending on the membrane lung properties and the gradient of carbon dioxide from the blood [8, 10–14].

Clinical studies have suggested that ECCO_2_R can improve right heart function during severe acute exacerbations of COPD (AE-COPD) [15], and also facilitate early extubation even in the most severe cases [13, 16]. Case-control studies by Braune et al. [14] and Del Sorbo et al. [12], demonstrated that avoiding intubation or allowing early extubation in acute AE-COPD was feasible, albeit with significant side effects, mainly bleeding and clotting.

Despite these encouraging results, two recent clinical trials, one for COPD and the other for ARDS produced discouraging results, questioning the underlying premise of ECCO_2_R in patients with COPD. The VENT-AVOID trial [17], a multicenter RCT examining the avoidance of IMV in acute exacerbation of COPD failed to show a significant difference in ventilator-free days at day 5. In another group of patients, the REST trial [18], a large multicenter RCT evaluating whether ECCO_2_R could improve outcomes in patients with acute hypoxemic respiratory failure by facilitating ultra-protective ventilation was stopped early for futility after enrolling 412 patients. Importantly, both trials had higher rates of adverse events in the active treatment arms. For example, REST had an incidence of intra-cranial hemorrhage of 4.5% in the ECCO_2_R arm compared to 0 in the control arm.

Notably, both trials used ECCO_2_R devices which were capable of only removing about 25% of the total CO_2_ production. The device used in both the VENT-AVOID and REST trials was the same low-flow system, the Hemolung Respiratory Assist System (ALung Technologies), operating at blood flow rates of up to approximately 550 mL/min. Furthermore, these systems used integrated-membrane blood pumps that are different from the centrifugal pump technology used in current ECMO systems. This latter factor may explain the increased adverse events in both trials with bleeding associated with thrombocytopenia, hemolysis and acquired von-Willebrand factor syndrome [19]. The only ECCO_2_R RCT (Xtravent trial in moderate to severe ARDS) to show a small potential clinical benefit, used a pumpless system, overcoming the problem of pump associated bleeding, and had a CO_2_ removal capacity almost twice as high as the devices used in the above-mentioned trials [20].

The X-COPD Trial (NCT03584295) was designed to overcome these two major drawbacks by deploying an ECCO_2_R device that uses conventional rotational blood pumps at relatively high blood flow rates (1–1.75 L/min) and thus has much higher CO_2_ elimination capacity. The goal was to evaluate whether early extubation assisted by ECCO_2_R would decrease 60-day mortality or severe disability in patients with severe AE-COPD requiring IMV in a three-center, randomized controlled trial.

## Material and methods

**Fig. 1:**
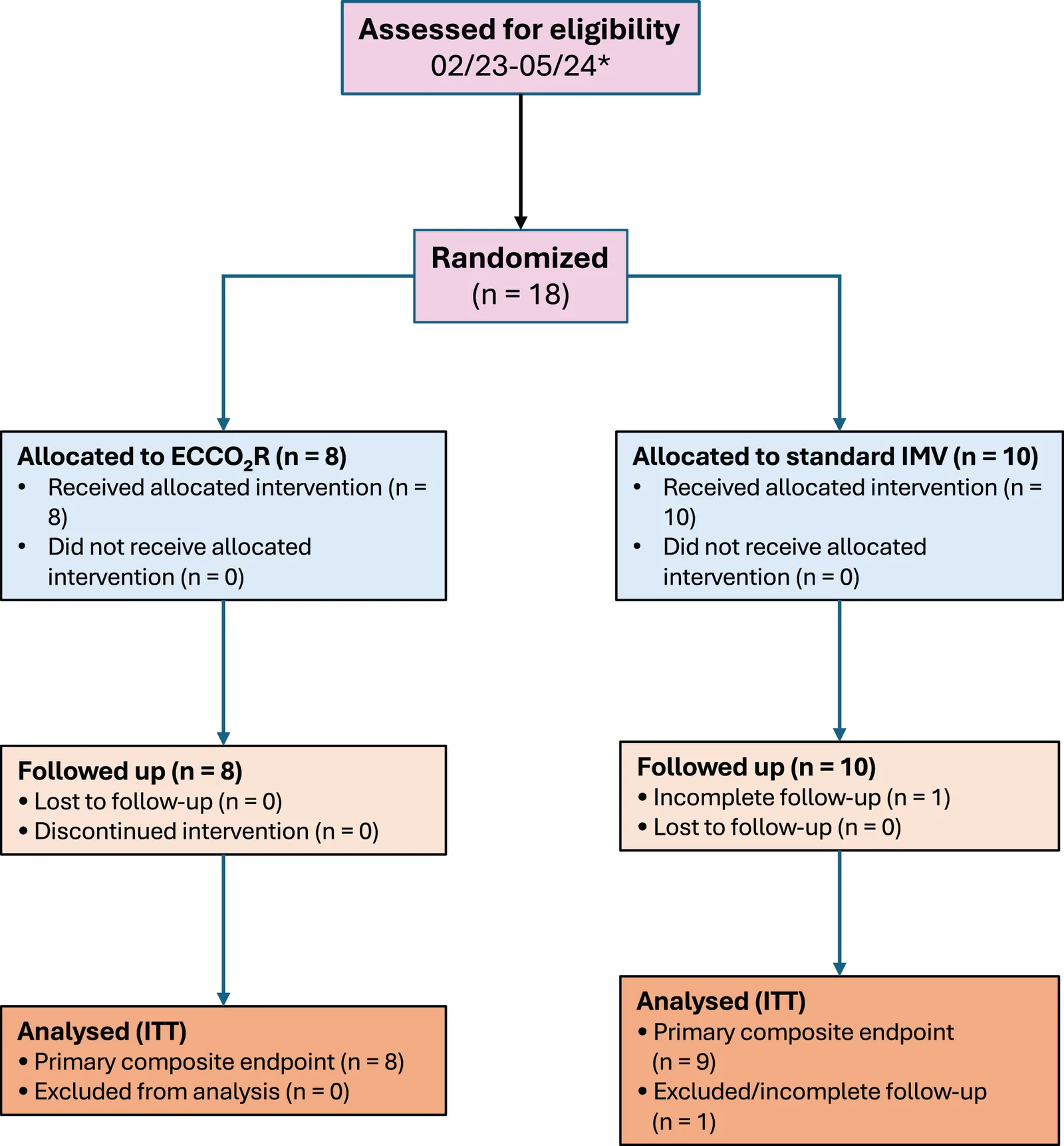
CONSORT flow diagram of the X-COPD trial. Eight patients were allocated to ECCO₂R-facilitated early extubation and ten to standard invasive mechanical ventilation (IMV). One patient in the IMV group had incomplete follow-up and could not be evaluated for the primary composite endpoint (death or severe disability at day 60), giving nine evaluable IMV patients. *the number of patients screened and excluded before randomization was not systematically recorded and is therefore not shown; this is noted in the study limitations

This study was performed in compliance with the international standard ISO 14155 (Clinical investigation of medical devices for human subjects - Good clinical practice), the European Medical Device Directive, the Declaration of Helsinki (with amendments) and any other applicable legal and regulatory requirements and approved by the ethical committee Z-303/2020. Clinical Trials No: NCT03584295, Eudamed-No: CIV-20–12-035287. Clinical Trials No: NCT03584295 gives some additional information about the trial. The flow of participants through the trial is shown in the CONSORT flow diagram (Fig. 1). Randomization to treatment arms was performed by a computer in a 1:1 ratio and stratified by recruitment center. The permuted block sizes randomly varied between 2, 4, and 6. The allocation sequence was concealed and administered centrally, and assignment was disclosed only after enrolment. Because ECCO_2_R involves a visible extracorporeal circuit, blinding of patients and treating clinicians was not possible; this open-label design and its potential for performance and ascertainment bias are addressed in the Limitations.

### Outcomes

The primary outcome was death or severe disability up to day 60 after randomization; severe disability was defined as confinement to bed and/or inability to wash or dress alone and/or need for long-term IMV at day 60. Functional status at day 60 was assessed by the treating study investigators at each participating center – in person while patients remained hospitalized and by structured contact with the patient or a surrogate after discharge; these assessors were not blinded to treatment allocation. Baseline functional status in the period before the acute exacerbation was documented at enrolment, and severe disability at day 60 was counted only when it represented new, persistent impairment rather than a pre-existing condition. As no patient met the definition of severe disability before randomization, all day-60 events reflect new disabilities.

Secondary outcomes were: mortality or severe disability up to day 180 after randomization; ventilator-associated pneumonia during intensive care unit (ICU) treatment according to the ATS 2016 clinical practice guideline [21]; reintubation rate; duration of total ventilatory support defined as days on IMV and/or non-invasive ventilation (NIV) or ECCO_2_R; thrombosis of major venous vessels during the treatment period; quality of life at day 60 and day 180 after randomization, measured with Severe Respiratory Insufficiency index (SRI) and EQ-5D-5L (the EQ-5D-5L utility index and the EQ visual analogue scale [EQ-VAS] are reported separately); tracheostomy up to day 60 after randomization; and severe bleeding. Thrombosis of major venous vessels was defined as newly diagnosed thrombosis of a central or major peripheral vein, including at cannulation sites, confirmed by Doppler ultrasonography or computed tomography. Severe bleeding was defined as bleeding that was fatal, occurred at a critical anatomical site, required transfusion of at least two units of packed red blood cells, or required a surgical or interventional procedure. Thrombotic and bleeding events were assessed by the treating physicians at each participating center and reviewed centrally by the coordinating investigators.

### Inclusion criteria

The study focused on patients who were difficult to manage with IMV. Minimum age of 18 years, known history of COPD with an acute exacerbation causing acute hypercapnic respiratory failure (defined as arterial pH < 7.35 with PaCO_2_ > 45 mmHg) and receiving IMV, potentially reversible cause of respiratory failure as determined by the treating physician (i.e. the acute exacerbation was judged likely to resolve with recovery of respiratory function toward the patient’s pre-exacerbation baseline, thereby excluding patients considered to be in an irreversible, end-stage or palliative situation), failed extubation attempt or extubation not possible within 24 hours after intubation according to the criteria of Boles et al. [22]. In this context, patients considered difficult to manage with invasive ventilation were specifically those in whom a first extubation attempt had failed, or in whom extubation was judged not feasible within 24 hours of intubation, despite optimized ventilator settings and pharmacological treatment of the exacerbation. Apart from the pH and PaCO_2_ thresholds defining acute hypercapnic respiratory failure (pH < 7.35 with PaCO_2_ > 45 mmHg), no additional ventilator-setting, blood-gas or clinical-marker thresholds were mandated for inclusion.

### ECCO_2_R

ECCO_2_R was applied in a standard configuration with either a double-lumen cannula (19–24 Fr double lumen cannula, either NovaPort twin, Avalon Elite or MC3 Crescent cannula) or two small single-lumen cannulae (15–19 Fr), allowing a blood flow rate between 1 and 1.75 L/min with the ILA active kit (Xenios, Fresenius, Germany). Systemic anticoagulation during ECCO_2_R was provided with continuous intravenous unfractionated heparin, targeting an activated partial thromboplastin time (aPTT) of approximately 1.5 times the upper limit of normal; aPTT was monitored at least once daily and the heparin dose adjusted accordingly. The same anticoagulation protocol was applied uniformly at all three participating centers. Daily weaning attempts from ECCO_2_R were made by turning the sweep gas flow off for 60–120 minutes. If the pH value remained above 7.35 and the respiratory drive was low, based on the overall clinical picture, the ECCO_2_R system was removed. While receiving invasive ventilation, patients in the ECCO_2_R group were managed according to the same ventilation principles as the control group, with PEEP titrated towards intrinsic PEEP (70–80% of PEEPi, or at least 5–8 cmH₂O) and particular attention to airflow limitation and dynamic hyperinflation; the additional CO_2_ clearance provided by ECCO_2_R allowed the intensity of ventilation to be reduced while these principles were maintained. By protocol, endotracheal extubation was prioritized over weaning from the extracorporeal circuit: once gas exchange was adequately supported, patients were extubated while remaining on ECCO_2_R, and daily sweep-gas-off trials to assess readiness for decannulation were undertaken only thereafter, in awake and spontaneously breathing patients. Liberation from ECCO_2_R while a patient was still sedated and receiving invasive ventilation was therefore not part of the treatment algorithm. For these sweep-gas-off trials, a low respiratory drive was defined pragmatically, on the basis of the overall clinical picture, as a spontaneous respiratory rate below approximately 25–30 breaths/min without signs of respiratory distress or excessive accessory-muscle use and with stable tidal volumes, while an arterial pH above 7.35 was maintained.

### Control Group

Patients in the control group received standard of care, which included IMV and guideline-adapted management of COPD patients, including attempts to extubate the patient and switch to NIV, as clinically appropriate. If extubation failed or was not clinically appropriate, tracheostomy could be performed according to the treating physician. IMV was performed according to recent guidelines [23] and airflow limitation/hyperinflation was given special attention. PEEP was set according to intrinsic positive end-expiratory pressure (PEEPi) measurements (70–80% of PEEPi) or at least between 5 to 8 cmH_2_O in any mode of mechanical ventilation. Breathing frequency was titrated down while measuring PEEPi and tidal volume/compliance. FiO_2_ was set to achieve peripheral oxygen saturation levels of 88–94%. All patients had to receive one extubation attempt before tracheostomy, if appropriate. Sedation and analgesia were performed according to local clinical standards, including short-acting agents; and level of sedation was monitored by RASS (Richmond Agitation and Sedation Score). Per protocol, rescue crossover to ECCO_2_R was permitted in the control group only for refractory, life-threatening respiratory acidosis (for example a persistent arterial pH below approximately 7.15 with worsening hypercapnia) despite optimized invasive ventilation and maximal medical therapy; these predefined rescue criteria were specified in advance. One control-group patient met these criteria and received ECCO_2_R as rescue therapy after randomization, continuing for 36 days; consistent with the intention-to-treat principle, this patient was analyzed in the IMV (control) group for all outcomes.

## Statistics

Sample size calculation was based on an absolute reduction of mortality of 10%, which was expected in the VV-ECCO_2_R treatment group. Since approximately 20% of all tracheostomized COPD patients in German weaning centres require long-term IMV for more than 28 days, an additional 10% reduction in severe disability was expected. The primary efficacy composite endpoint was death or severe disability at day 60 after randomization (defined as death by day 60 or before discharge from hospital at any time to the end of data collection). Severe disability was defined as confinement to bed and/or inability to wash or dress alone and/or need for long-term IMV.

A sample size of 192 patients in a 1:1 ratio to experimental or control group yielded an 80% power to detect a 40% relative risk reduction in the primary composite outcome of death or severe disability at 60 days, from 50% in the control group (30% mortality and 20% severe disability) to 30% in the investigational group (20% mortality and 10% severe disability) at a two-sided alpha of 0.05, accounting for 5% loss to follow-up. The analysis was performed on the full analysis set (all randomized patients) according to the intention-to-treat principle. Because the primary composite endpoint could not be ascertained in one IMV-group patient with incomplete follow-up, this comparison was in practice an available-case analysis (evaluable patients 0/8 vs 3/9); to gauge the influence of this single missing observation, a simple sensitivity analysis assigning that patient in turn to the event (worst case) and to the non-event (best case) category was performed and is reported in the Results. Categorical endpoints were compared using Fisher’s exact test, which is more appropriate than the originally planned chi-squared test given the small, realized sample size. The analysis was performed according to the intention to treat principle in the full analysis population using a Chi-squared test. An interim analysis was planned after 134 patients (70%) reached the 60-day follow-up to verify the assumptions of the sample size calculation. If the assumption of the sample size calculation was verified, the recruitment would stop at the initially targeted sample size of 192. These effect-size assumptions were derived from the physiological and observational ECCO_2_R literature and from expert consensus available when the trial was designed, before publication of the VENT-AVOID and REST trials. In retrospect, and in light of the more modest treatment effects subsequently reported in those trials (including the change of the VENT-AVOID primary endpoint from ventilator-free days at day 60 to day 5), an assumed absolute mortality reduction of 10% is likely to have been optimistic, and the resulting target sample size should therefore be regarded as a best-case estimate.

Between-group comparisons of continuous variables were performed using Welch’s two-sample t-test. Categorical variables were compared using Fisher’s exact test. Effect estimates are reported as mean differences or risk differences with corresponding two-sided 95% confidence intervals. Because of the small sample size, 95% confidence intervals for risk differences were calculated using the Newcombe hybrid-score method, which is consistent with the exact tests used for hypothesis testing. A two-sided *p*-value < 0.05 was considered statistically significant. Because the trial was terminated early after enrolment of only 18 of the planned 192 patients, it is severely underpowered for death, severe disability, and every other clinical outcome; accordingly, all analyses of clinical endpoints are regarded as exploratory and hypothesis-generating, and the reported *p*-values and confidence intervals are descriptive rather than confirmatory tests of efficacy. Device-free days were a post hoc, exploratory outcome and were not prespecified in the protocol or statistical analysis plan; they are reported as such. Device-free days were defined as the number of days, within the first 29 days after randomization, on which a patient was both alive and simultaneously free of invasive mechanical ventilation, non-invasive ventilation and ECCO_2_R. A day on which a patient received any of these forms of support, or on which the patient was not alive, was counted as not device-free (contributing zero device-free days). Day 29 (rather than day 28 or 30) was chosen so as to capture status across the first 28 completed days after randomization (the day of randomization plus 28 days), reflecting the widely used ~28-day threshold that separates prolonged/long-term invasive ventilation from shorter-term support in weaning populations. For patients receiving more than one form of support simultaneously (for example ECCO_2_R overlapping with invasive or non-invasive ventilation), the overlapping day was counted only once and as not device-free. Patients were analyzed in the group to which they were randomized; accordingly, the ECCO_2_R days of the single IMV-group patient who crossed over to ECCO_2_R under the predefined rescue criteria were counted as support (non-device-free) days within the IMV arm. Because device-free days depend on survival, patients who died within the 29-day window contributed device-free days only up to death and zero thereafter; 17 of the 18 randomized patients were alive at day 29 and contributed to this analysis (Supplemental Figure 4).

## Results

The study was discontinued by the sponsor for financial reasons after 18 patients were randomized in three centers. Patients were assessed from 02/23–05/24 for eligibility. 18 met the inclusion criteria and were randomized (Fig. 1).

### Study population

**Fig. 2:**
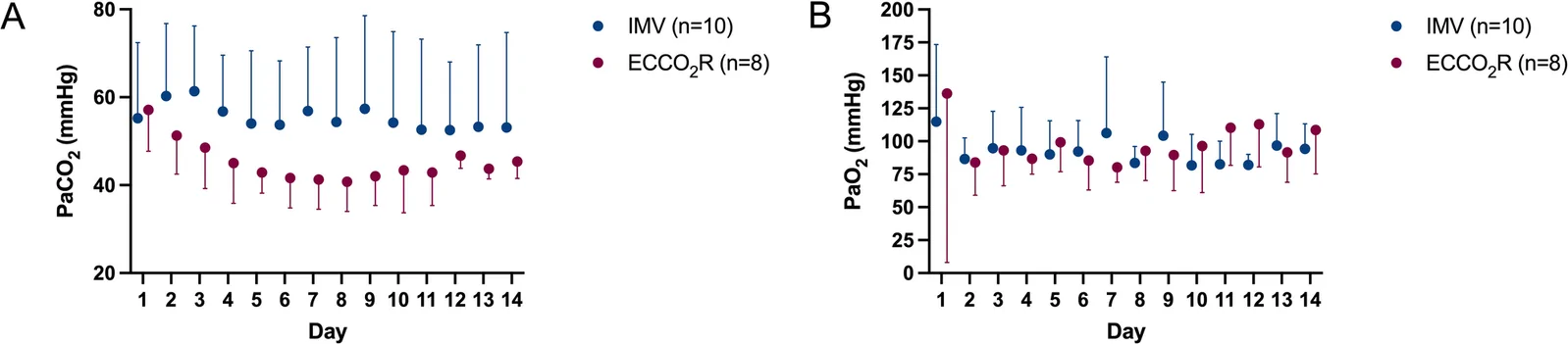
Temporal progression of PaCO2 (**A**) and PaO2 (**B**) during the initial treatment phase up to day 14. Error bars demonstrate the standard deviation

**Fig. 3:**
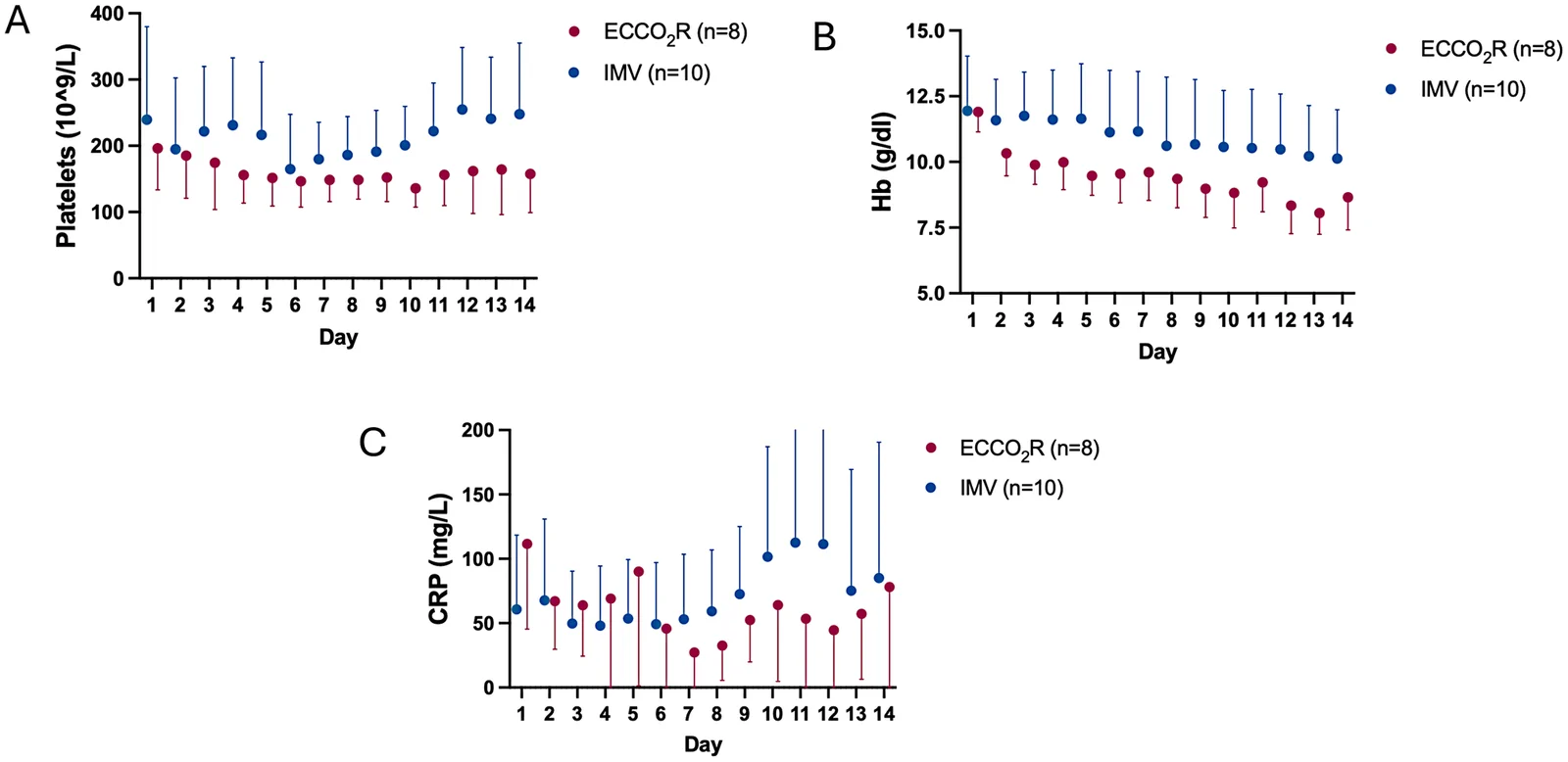
Temporal progression of hemoglobin (hb, **A**), platelets (**B**) and CRP (**C**) during the initial treatment phase up to day 14. Error bars demonstrate the standard deviation

**Fig. 4:**
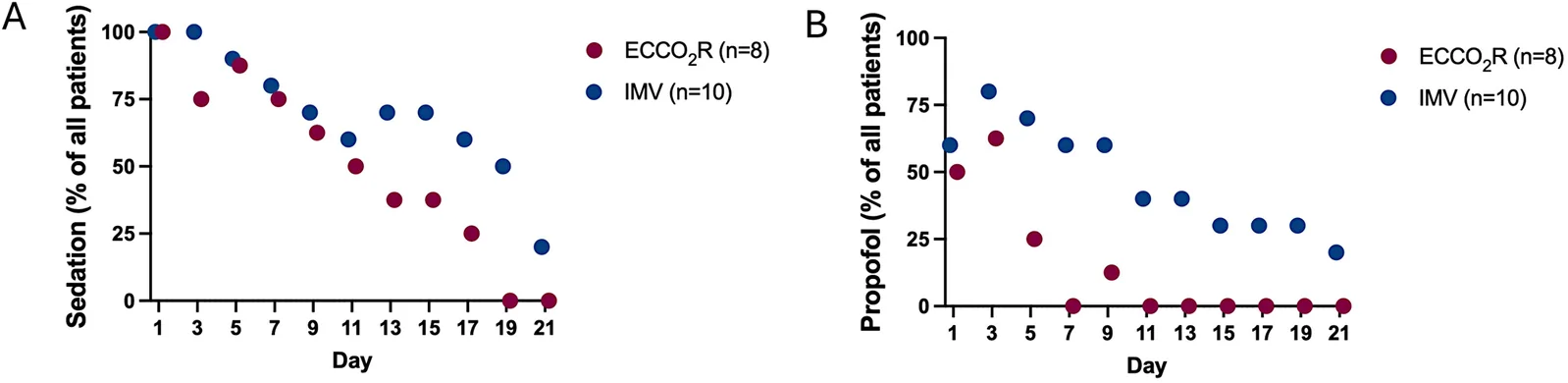
Temporal progression of the proportion of patients undergoing sedation in general(**A**) and of propofol (**B**). Error bars demonstrate the standard deviation

**Fig. 5:**
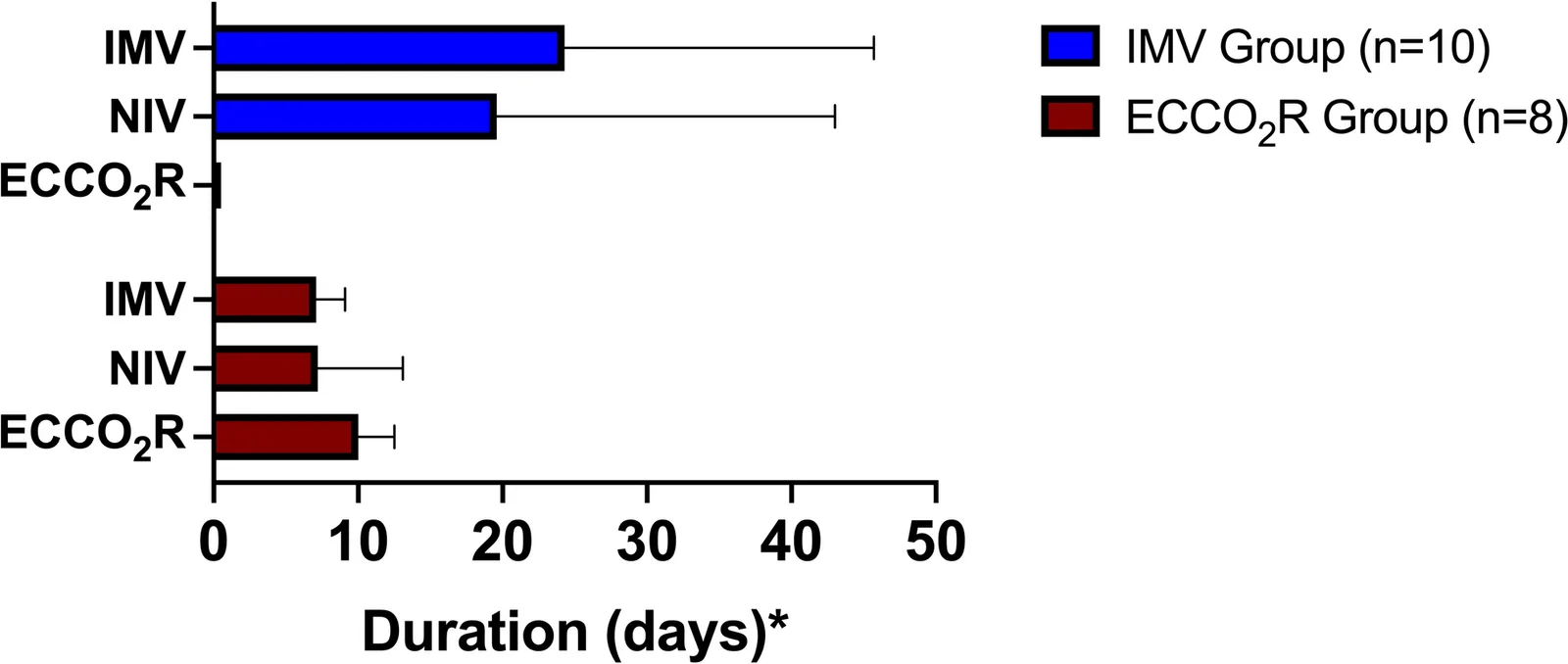
Duration of invasive mechanical ventilation (IMV), non-invasive ventilation (NIV) or extracorporeal CO2 elimination (ECCO2R). Of note ECCO2R treatment may occur at the same time as IMV or NIV. Error bars demonstrate the standard deviation. One patient in the IMV group received ECCO2R due to an otherwise uncompensated hypercapnia and survived. ^*^ECCO_2_R and IMV or NIV may occur at the same time

A total of 18 randomized patients were included in the analysis, eight treated with ECCO₂R and 10 treated with IMV. All 18 randomized patients (8 ECCO_2_R, 10 IMV) were analyzed in the group to which they were randomized (full analysis set) and are presented in Table 1 and Figs. 1, 2, 3, 4 and 5; as detailed in the Statistics section, the primary composite endpoint was evaluable in 8 ECCO_2_R and 9 IMV patients, so that this comparison represents an available-case analysis rather than a complete-case intention-to-treat analysis. For the primary composite endpoint, one patient in the IMV group with incomplete follow-up could not be evaluated, yielding nine evaluable IMV patients (Table 2); where a single additional outcome component could not be assessed the denominator was reduced accordingly and reported as a missing value. The single IMV-group patient who could not be evaluated for the primary composite endpoint was lost to follow-up before the day-60 assessment; this patient is shown in the CONSORT diagram (Fig. 1) and is the reason the evaluable IMV denominator is nine rather than ten. Baseline characteristics are summarized in Table 1. Mean age (62.9 ± 6.2 vs. 65.6 ± 6.5 years) and sex distribution were similar between ECCO_2_R and IMV groups, respectively. Patients in the ECCO₂R group had lower mean body weight (62.5 ± 15.2 vs. 84.9 ± 23.4 kg) and body mass index (22.3 ± 3.6 vs. 29.2 ± 5.7 kg/m^2^). Smoking status and long-term oxygen therapy use were similar between groups. Pulmonary function parameters prior to ICU admission were comparable between groups (mean FEV₁ 1.2 ± 0.5 vs. 1.3 ± 0.7 L/s; vital capacity 2.7 ± 1.1 vs. 2.3 ± 1.1 L) (Table E1). No patient had a severe disability prior to randomization. Baseline severity of illnesses are summarized in Table 1 and Table E1, whereas blood gas values are given from day 1–14 in Fig. 2.

**Table 1:** Baseline characteristics

	ECCO2R (N = 8)	IMV (N = 10)
**Age (years)**		
Mean ± SD	62.9 ± 6.2	65.6 ± 6.5
Median (Min; Max)	61.5 (57.0; 76.0)	65.5 (57.0; 76.0)
**Sex, n (%)**		
Female	4 (50.0)	5 (50.0)
Male	4 (50.0)	5 (50.0)
**Body height (cm)**		
Mean ± SD	167.0 ± 14.0	169.4 ± 13.2
Median (Min; Max)	167.5 (150.0; 187.0)	167.5 (155.0; 190.0)
**Body weight (kg)**		
Mean ± SD	62.5 ± 15.2	84.9 ± 23.4
Median (Min; Max)	55.3 (48.0; 87.0)	85.0 (60.0; 120.0)
**BMI**		
Mean ± SD	22.3 ± 3.6	29.2 ± 5.7
Median (Min; Max)	22.5 (16.6; 26.9)	27.6 (22.3; 39.0)
**Smoking status**		
**Smoker**	**6 (75.0)**	**7 (70.0)**
Ex-Smoker	2 (25.0)	3 (30.0)
Never Smoker	0 (0.0)	0 (0.0)
**Long-term oxygen-therapy**		
Yes	1 (12.5)	3 (33.3)
No	7 (87.5)	6 (66.7)
**Peripheral vascular disease**		
Yes	1	1
No	7	9
**Diabetes mellitus**		
Yes	0	2
No	8	8
**Moderate or severe renal disease**		
Yes	1	0
No	7	10
**P/F ratio (mmHg) before randomization**		
Mean ± SD	279.6 ± 104.9	261.8 ± 117.2
**Onset of IMV before randomization (h)**		
Mean ± SD	46.1 ± 18.7	38.8 ± 22.8

**Table 2:** Major outcomes

	**ECCO**_**2**_**R** (*N* = 8)	IMV* (*N* = 9)
**Pneumonia occurring during ICU treatment**		
No	8 (100.0%)	5 (62.5%)
Yes	0 (0.0%)	3 (37.5%)
**Mortality and/or severe disability at day 60**		
No	8 (100.0%)	6 (66.7%)
Yes	0 (0.0%)	**3 (33.3%)**
**Mortality at day 60**		
No	8 (100.0%)	8 (88.9%)
Yes	0 (0.0%)	**1 (11.1%)**
**Severe Disability at day 60**		
No	8 (100.0%)	6 (75.0%)
Yes	0 (0.0%)	**2 (25.0%)**
missing values	0	1
**SRI at day 60**		
Mean ± SD	71.6 ± 15.8	60.3 ± 19.3
**EQ-5D-5L: VAS**		
Mean ± SD	66.3 ± 14.1	55.0 ± 21.8

*One patient randomized to the IMV group was excluded from the outcome analysis owing to incomplete follow-up, yielding *N* = 9; where a further outcome could not be assessed the denominator is reduced accordingly and reported under “missing values”. The analysis followed the full-analysis-set (intention-to-treat) principle, including all randomized patients for whom the respective outcome was available; because the primary composite endpoint could not be ascertained in one IMV-group patient, that comparison is an available-case analysis (0/8 vs 3/9), and a sensitivity analysis for the missing patient is reported in the main text. Bold values denote the number of patients experiencing the event (i.e. the outcome of interest). Risk differences with two-sided 95% confidence intervals (Newcombe hybrid-score method) and exact *p*-values for each comparison are given in the main text. To make the denominators explicit: eight ECCO₂R and nine IMV patients were evaluable for the composite endpoint, mortality, and severe disability, whereas pneumonia during ICU treatment could be assessed in eight ECCO₂R and eight IMV patients (one further IMV patient had missing data), so the pneumonia proportion in the IMV group is 3/8 (37.5%). Within each outcome the tabulated categories sum to the number of evaluable patients, and patients without an available assessment are reported as missing rather than assumed event-free

### Clinical outcomes

At day 60, the composite endpoint of mortality and/or severe disability was observed in 0 of 8 patients treated with ECCO₂R and in 3 of 9 patients (33.3%) treated with IMV (risk difference −33.3%; 95% CI −64.6% to 5.5%; *p* = 0.21) (Table 2). In a sensitivity analysis addressing the single IMV-group patient with incomplete follow-up, assigning this patient to the event category (worst case) gave a composite event rate of 4/10 (40.0%) in the IMV group versus 0/8 with ECCO_2_R, whereas assigning them to the non-event category (best case) gave 3/10 (30.0%) versus 0/8; the direction of the between-group difference was unchanged under both assumptions. Sixty-day mortality occurred in 1 out of 9 IMV-treated patients (11.1%) and in none of the ECCO₂R-treated patients (risk difference −11.1%; 95% CI −43.5% to 22.6%; *p* = 1.00). Severe disability at day 60 was reported in 2 of 8 evaluable IMV-treated patients (25.0%) and in none of the ECCO₂R-treated patients (risk difference −25.0%; 95% CI −59.1% to 12.0%; *p* = 0.47), while one patient in the IMV group died before the day-60 disability assessment. Ventilator associated pneumonia during ICU treatment occurred in none of the ECCO₂R-treated patients and in 3 of 8 evaluable IMV-treated patients (37.5%) (risk difference −37.5%; 95% CI −69.4% to 2.7%; *p* = 0.20). Reintubation occurred in 2 of 8 patients (25.0%) treated with ECCO₂R and in 3 of 8 IMV-treated patients (37.5%) (risk difference −12.5%; 95% CI −49.1% to 29.1%; *p* = 1.00) (Table E3).

Health-related quality of life, assessed with the Severe Respiratory Insufficiency (SRI) questionnaire and the EQ-5D-5L, and total hospital treatment costs were pre-specified secondary outcomes. After premature termination, the sample was too small for any meaningful health-economic analysis. EQ-5D-5L and SRI at day 60 are given in Table 2 and the online supplement (more detailed data).

### Physiological parameters

Temporal changes in respiratory and physiological parameters are presented in Figs. 1, 2, 3, 4, 5 and Supplemental Figures 1–3. In ECCO₂R-treated patients’ improvement in ventilatory and gas exchange parameters appeared to occur sooner and be more sustainable, compared to patients treated with IMV alone. Throughout the initial 14-day treatment phase, the partial pressure of arterial carbon dioxide (PaCO₂) values were higher in the IMV group (approximately 55–65 mmHg) compared to the ECCO₂R group (approximately 40–50 mmHg), documenting the efficacy of the extracorporeal circuit. The partial pressure of arterial oxygen (PaO₂) levels remained comparable between the two groups over the same period, with values generally ranging between 75 and 130 mmHg in both arms (Fig. 2). Hemoglobin levels in the ECCO₂R group were lower than in the IMV group across most time points (approximately 12–13 g/dL vs. 9–11 g/dL). Platelet counts were also higher in the control group throughout the observation period, while both groups showed platelet levels within a broadly acceptable range (approximately 150–300 × 10^9^/L). CRP values were elevated in both groups early in the treatment course and showed a gradual decline over time, consistent with resolving systemic inflammation, without a clear differential between the two groups (Fig. 3).

All patients received sedation on day 1. The ECCO₂R group reached near-zero sedation by approximately day 13, compared to the IMV group, in which sedation weaning was more gradual and prolonged – beyond day 21. The use of propofol followed a similar pattern: it was discontinued earlier in the ECCO₂R group (by approximately day 9), while the IMV group continued to receive propofol through day 21 in a proportion of patients (Fig. 4).

### Duration of ventilatory support

The duration of invasive mechanical ventilation was shorter in the ECCO₂R group than in the IMV group (7.1 ± 2.0 vs. 24.3 ± 21.4 days; mean difference −17.2 days; 95% CI −33.5 to −0.9; *p* = 0.043). The duration of NIV did not differ significantly between groups (7.2 ± 5.9 vs. 19.6 ± 23.4 days; mean difference −12.4 days; 95% CI −30.7 to 5.9; *p* = 0.16). Median duration of ECCO₂R therapy was 9.5 days (range 7.0–14.0) (Fig. 5, Table E2). The ECCO₂R group had substantially more device-free days at day 29 compared to the IMV group, defined as the number of days alive and free of ECCO₂R, IMV and NIV. Patients in the ECCO₂R group had 17 ± 4 device-support-free days at day 29 compared with 8 ± 7 days in the IMV group (*p* = 0.011). Notably, ECCO₂R support could overlap in time with both IMV and NIV. Because the IMV group included one markedly prolonged outlier (maximum 59 days) and its duration distribution was right-skewed, these duration outcomes should be interpreted primarily on the basis of medians and descriptive data rather than means and *p*-values. The median duration of invasive ventilation was 7.0 days (range 4.0–10.0) in the ECCO_2_R group versus 16.0 days (range 3.0–59.0) in the IMV group, and the median duration of NIV was 4.0 (2.0–15.0) versus 7.0 (1.0–55.0) days (Table E2). One patient randomized to the IMV group met the predefined rescue criteria and received ECCO_2_R (for 36 days); consistent with the intention-to-treat approach, this patient was retained in the IMV group for all analyses and contributed to the long right tail of the IMV duration distribution, further supporting a cautious, median-based interpretation of these outcomes.

### Safety

Severe bleeding complications were reported in 1 of 8 ECCO₂R-treated patients (12.5%) and in none of the IMV-treated patients (risk difference 12.5%; 95% CI −21.5% to 47.1%; *p* = 0.47) (Table E4). The only severe bleeding complication that occurred was a retroperitoneal hematoma which resolved over time. One thrombosis was recorded in the ECCO_2_R device group in the right jugular vein, without requiring any further therapeutic measures. No device failures or circuit-related adverse events were reported. Anemia and thrombocytopenia occurred in 2 patients of each group.

## Discussion

In this small randomized controlled trial of patients with severe AE-COPD requiring IMV, which was stopped due to financial reasons by the study sponsor, ECCO_2_R-facilitated early extubation was feasible and was not associated with any unexpected safety signal. Due to the very small sample size, the observed between-group differences were accompanied by wide confidence intervals.

Although the study was clearly underpowered to assess differences in clinical outcomes, all major clinical outcomes pointed in the same direction. Patients treated with ECCO_2_R experienced earlier extubation, shorter duration of IMV, fewer episodes of pneumonia and shorter length of hospital stay. No death or severe disability occurred in the ECCO_2_R group at day 60 compared to 3 out of 9 in the standard care group. Of note, the patients treated with ECCO_2_R had a lower body mass index, a baseline imbalance that limits causal inference; although a low body mass index has been associated with worse outcomes in COPD [24].

The absence of ICU-acquired pneumonia in the ECCO_2_R group suggests that shortening the duration of IMV may have downstream effects on infectious morbidity. Consistent with this hypothesis, patients assigned to ECCO_2_R demonstrated a lower combined endpoint of mortality or severe disability at day 60, driven primarily by reductions in functional impairment rather than mortality alone. Importantly these data are in line with the first RCTs decades ago, demonstrating the superiority of NIV vs. invasive mechanical ventilation, especially in terms of infectious complications [25, 26].

The earlier discontinuation of sedation in the ECCO₂R group may represent an additional mechanism which could contribute to improved outcomes. Because extracorporeal CO₂ removal supported spontaneous breathing and enabled extubation, sedation and propofol could be withdrawn several days earlier than in the IMV group. Earlier cessation of sedation facilitates earlier mobilization, active participation in physiotherapy and functional recovery, and is associated with less ICU-acquired weakness and delirium; these effects could plausibly have contributed to a possible lower rate of severe disability in the primary composite endpoint. This potential pathway linking earlier sedation withdrawal to improved functional recovery warrants dedicated evaluation in adequately powered future trials. Furthermore, although the number of patients was very small and no firm conclusions can be drawn, health-related quality-of-life scores at day 60 (the SRI summary score and the EQ-5D-5L, with the utility index and the EQ-VAS reported separately in the online supplement) were numerically higher in the ECCO_2_R group; given the small sample and the inconsistent direction of some indices over time, we refrain from concluding that either group had a better quality of life.

Although this study is clearly underpowered with respect to clinically important outcomes, it is interesting to speculate on why these results might be different from previous studies. The current ECCO_2_R evidence for acute respiratory failure due to COPD originates from one large trial, using a device which was only capable of removing about 25% of the total CO_2_ production, while using an extracorporeal technique, which may substantially induce bleeding triggered by thrombocytopenia, hemolysis and acquired von-Willebrand factor syndrome [19]. These technical differences are substantial. The increased CO_2_ removal capacity in the current study (at least two times higher) allows for a greater reduction in the intensity of ventilation [13, 27], which could reduce gas trapping, a major cause of morbidity in hyperinflated patients with COPD. As well, we used an ECCO_2_R device with classical diagonal/centrifugal pumps, which may be associated with lower complication rates. The CO_2_ removal capacity was even higher than that used in the Xtravent trial, which used a pumpless technique. The Xtravent trial was the only ECCO_2_R RCT demonstrating a potential clinical benefit in moderate to severe ARDS. Our data are in accord with the hypothesis that the failure of ECCO_2_R may be a technological failure rather than a conceptual failure as recently pointed out by Barbic et al., and Slutsky et al. [28, 29].

When interpreted in the context of the VENT-AVOID trial, our findings appear complementary rather than contradictory. VENT-AVOID did not show a significant difference in its primary endpoint of ventilator-free days at day 5, but this overall neutral result was driven largely by the non-invasive ventilation stratum, in which ECCO_2_R was used to avoid intubation and in which mortality was in fact higher. In the pre-specified invasive-ventilation (weaning) stratum, by contrast, ECCO_2_R was associated with numerically more ventilator-free days at day 5 (median 2 vs. 0.25 days), comparable mortality (17% vs. 15%) and no increase in ICU or hospital length of stay. The accompanying editorial reached a similar conclusion, arguing that the potential benefit of ECCO₂R lies in weaning intubated patients rather than in avoiding intubation in patients receiving non-invasive ventilation [30]. Because the X-COPD trial enrolled exclusively intubated patients requiring weaning, it directly addresses that population and lends further support to a possible role for ECCO_2_R in facilitated weaning. The longer duration of invasive ventilation in our cohort than in VENT-AVOID is most plausibly explained by differences in the enrolled populations: X-COPD included only patients who had already failed, or were ineligible for extubation within 24 hours of intubation and thus represented a more severe, weaning-failure phenotype, whereas the invasive (weaning) stratum of VENT-AVOID enrolled patients earlier in their ventilatory course. In our cohort, invasive ventilation extended well beyond day 5 in essentially all patients (mean duration of invasive ventilation 7.1 days even in the ECCO₂R group), so ventilator-free days at day 5 would be close to zero in both arms and largely uninformative; we therefore additionally report device-free days at day 29 as a post hoc, exploratory outcome. Notably, the mean duration of ECCO₂R support in the present trial (approximately 10 days) was considerably longer than in earlier studies (approximately 4.6 days in VENT-AVOID and 5 days in the cohort reported by Del Sorbo and colleagues), which may reflect a deliberate strategy of maintaining extracorporeal CO₂ removal until sustained liberation from invasive ventilation had been achieved. Finally, because ECCO₂R and the early-extubation strategy were applied together, the present design cannot determine whether the apparent benefit is attributable to the device itself, to the early-extubation strategy, or to a combination of the two; this question should be addressed explicitly in future trials.

Our results are in line with previous non-randomized controlled trials [12–14] demonstrating the feasibility of early extubation with ECCO_2_R. The safety of ECCO_2_R in this trial, which is a crucial point in extracorporeal treatment, especially when using relatively low blood flow rates [31–33], was high, also likely related to the expertise of the three centres. This is evident, among other things, in the slow increase in sweep gas flow rate so as not to eliminate CO_2_ too quickly (Supplemental Figure 1), and in the relatively stable course of the coagulation parameters over time, aiming at an activated partial thromboplastin time (aPTT) of 1.5-times the upper limit. The PaCO_2_ decline was faster compared to the conventional group, but concerningly low levels of CO_2_ were clearly avoided. Furthermore, the sometimes-observed deterioration of the oxygenation in COPD patients treated with ECCO_2_R was not evident in this clinical trial, perhaps attributable to the physiological PaCO_2_ values achieved. This preserved oxygenation may also relate to the comparatively high blood flow rates (1–1.75L/min) and greater CO_2_ removal capacity used here, relative to the low-flow techniques employed in earlier studies. Nonetheless, given the very small sample size, we cannot exclude that a clinically relevant deterioration in oxygenation was simply not detected in this cohort, and this observation should therefore be regarded as hypothesis-generating.

### Limitations

This study has limitations. Most importantly, the very small sample size limits statistical power and results in imprecise effect estimates. Although randomization is intended to balance prognostic factors, several clinically relevant baseline differences were nonetheless present—most notably a lower body mass index in the ECCO_2_R group and a higher rate of long-term oxygen therapy in the IMV group, which may have influenced the observed outcomes and should be borne in mind when interpreting the between-group differences. In addition, the study was performed in expert centres, which may limit generalizability to other institutional settings with different expertise in extracorporeal support. The trial was unblinded and, although extubation and weaning decisions followed pre-specified criteria (Boles et al.), no rigidly protocolized algorithm for discontinuation of invasive ventilation was applied; in an open-label design this may have introduced performance or ascertainment bias in favor of the intervention, and future adequately powered trials should incorporate even more standardized weaning and extubation protocols. Taken together, these limitations preclude causal inference and underscore the need for adequately powered randomized controlled trials. Of note, although the sample size was small, recruitment went well, randomizing 18 patients in less than 12 months, pointing out the feasibility of a larger RCT with more centers. In addition, the number of patients screened and excluded before randomization was not systematically recorded and is therefore not shown in the CONSORT flow diagram (Fig. 1); this limits assessment of the representativeness of the enrolled cohort and should be captured prospectively in future trials. Finally, given the very small sample and a right-skewed control-group distribution influenced by a single long-stay outlier, the duration-of-ventilation outcomes should be regarded as descriptive and interpreted with caution, and the post hoc device-free-days analysis should be viewed as hypothesis-generating.

## Conclusions

In summary, in patients with severe AE-COPD who required IMV due to acute hypercapnic respiratory failure, the use of modern ECCO₂R devices, with sufficient CO_2_ removal capacity, enabled early extubation and may be associated with a shorter duration of IMV and NIV, and, in these exploratory analyses, with numerically fewer infectious complications and better functional outcomes and quality of life at day 60. Given the small sample and premature termination, these observations are hypothesis-generating. Together with mechanistic considerations they provide a rationale for an ECCO_2_R-enabled early extubation strategy and justify larger randomized multicentre trials to define its role in the management of ventilated COPD patients.

## Electronic supplementary material

Below is the link to the electronic supplementary material.

Supplementary material 1

## Data Availability

All data supporting the findings of this study are available within the paper and its Supplementary Information.

## Declarations

### List of collaborators

Antonio Pesenti (EMERGENCY ONG ETS, Milan, Italy antonio.pesenti@unimi.it., Università degli Studi di Milano, Milan, Italy), Mike Lars Broman (ECMO Center Karolinska, Department of Pediatric Perioperative Medicine and Intensive Care, Karolinska University Hospital, Stockholm, Sweden), Niall Ferguson (Toronto General Hospital, Head of Critical Care, UHN & Mount Sinai, Niall.ferguson@uhn.ca) and Christophe Guervilly (Service de Médecine Intensive-Réanimation, Hôpital Nord, Assistance Publique-Hôpitaux de Marseille, Marseille, France, christophe.guervilly@ap-hm.fr).
