# Supplementary material for: Extracorporeal CO2 elimination for acute exacerbation of severe COPD requiring invasive mechanical ventilation: a randomized controlled trial (the X-COPD trial)
Source: Crit Care. 2026 Sep 3;30:458. doi: 10.1186/s13054-026-06300-6 (PMC13543343; doi:10.1186/s13054-026-06300-6)
Supplement: Supplementary file 1 [file 13054_2026_6300_MOESM1_ESM.docx]

**Online Supplement**

**Table E1: Lung function testing before ICU admission**

|  | ECCO_2_R | IMV | Total |
| --- | --- | --- | --- |
|  | (N=8) | (N=10) | (N=18) |
| **FEV1 (L/s)** |  |  |  |
| n | 6 | 9 | 15 |
| Mean | 1.2 | 1.3 | 1.3 |
| SD | 0.5 | 0.7 | 0.6 |
| Median | 1.1 | 1.2 | 1.1 |
| Minimum | 0.7 | 0.6 | 0.6 |
| Maximum | 2.2 | 2.4 | 2.4 |
|  | | | |
| **Vital Capacity (L)** |  |  |  |
| n | 6 | 10 | 16 |
| Mean | 2.7 | 2.3 | 2.5 |
| SD | 1.1 | 1.1 | 1.1 |
| Median | 2.4 | 2.2 | 2.3 |
| Minimum | 1.5 | 1 | 1 |
| Maximum | 4.4 | 4.1 | 4.4 |

**Table E2: Duration of invasive mechanical ventilation (IMV), non-invasive mechanical ventilation (NIV) and ECCO2R**

|  | **ECCO_2_R** | **IMV** |
| --- | --- | --- |
| **Duration of IMV (days)** |  |  |
| Mean ± SD | **7.1 ± 2.0** | **24.3 ± 21.4** |
| Median (Min; Max) | 7.0 (4.0; 10.0) | 16.0 (3.0; 59.0) |
|  |  |  |
| **Duration of NIV (days)** |  |  |
| Mean ± SD | **7.2 ± 5.9** | **19.6 ± 23.4** |
| Median (Min; Max) | 4.0 (2.0; 15.0) | 7.0 (1.0; 55.0) |
|  |  |  |
| **Duration of ECCO2R (days)** | |  |
| Mean | 10.0 ± 2.5 | 36.0 ± - |
| Median (Min; Max) | 9.5 (7.0; 14.0) | 36.0 (36.0; 36.0) |

**Table E3: Reintubation rate**

| **Reintubation rate** | **ECCO_2_R (N=8)** | **IMV**  **(N=9)*** |
| --- | --- | --- |
| No reintubation | 6 (75.0%) | 5 (62.5%) |
| Patients intubated once | 2 (25.0%) | 3 (37.5%) |
| missing values | 0 | 1 |

* IMV percentages are calculated among the eight evaluable patients, with the remaining patient shown under 'missing values'

**Table E4: Severe Bleeding events (SAE)**

| **Severe bleeding** | | **ECCO_2_R (N=8)** | | **IMV (N=9*)** |  |
| --- | --- | --- | --- | --- | --- |
| No | | 7 (87.5%) | | 8 (100.0%) |  |
| Yes | | **1 (12.5%)** | | 0 (0.0%) |  |
| missing values | 0 | | 1 | | |

* IMV percentages are calculated among the eight evaluable patients, with the remaining patient shown under 'missing values'

**Analysis of AEs**

Most reported AEs by system organ classes (SOCs) were:

- gastrointestinal disorders (4 patients, 50.0% in ECCO_2_R, 8 patients, 80.0% in IMV),
- infections and infestations (4 patients, 50.0% in ECCO_2_R, 7 patients, 70.0% in IMV) and
- psychiatric disorders (7 patients, 87.5% in ECCO_2_R, 4 patients, 40.0% in IMV).

Most reported AEs on preferred term (PT) level were:

- constipation (2 patients, 25.0% in ECCO_2_R, 5 patients, 50.0% in IMV),
- delirium (4 patients, 50.0% in ECCO_2_R, 3 patients, 30.0% in IMV),
- hypertension (3 patients, 37.5% in ECCO_2_R, 3 patients, 30.0% in IMV),
- anaemia (2 patients, 25.0% in ECCO_2_R, 2 patients, 20.0% in IMV),
- dysphagia (2 patients, 25.0% in ECCO_2_R, 2 patients, 20.0% in IMV),
- thrombocytopenia (2 patients, 25.0% in ECCO_2_R, 2 patients, 20.0% in IMV).

#### **Analysis Serious Adverse Events (SAEs)**

4 patients (50.0%) in the ECCO_2_R group and 6 patients (60.0%) in the IMV group reported at least one SAE.

Most reported SAEs were:

- infections and infestations (2 patients (25.0%) in ECCO_2_R, 4 patients (40.0%) in IMV) and
- psychiatric disorders (0 patients in ECCO_2_R, 3 patients (30.0%) in IMV).

SAEs on preferred term (PT) level occurring in at least 2 patients in total were:

- pneumonia (1 patient (12.5%) in ECCO_2_R, 3 patients (30.0%) in IMV),
- delirium (0 patients in ECCO_2_R, 3 patients (30.0%) in IMV)

Quality of life

**Course of SRI**

The Severe Respiratory Insufficiency Questionnaire (SRI) is a well validated and specific HRQoL measurement tool for the condition of chronic respiratory failure. The SRI questionnaire contains 49 items and seven subscales added into a summary score (range 0-100) with higher scores indicating a better HRQoL.

Overall, HRQoL was rated better or equally good in ECCO_2_R patients compared IMV patients for most subscales and the summary scale. HRQoL was also rated better at day 60 compared to day 180 in both groups.

At day 60, a mean ± SD of 71.6 ± 15.8 ranging from 46.8 to 93.2 (median 73.2) was documented for the ECCO_2_R group (n=8) and of 60.3 ± 19.3 ranging from 35.1 to 88.9 (median 66.0) in the IMV group (n=7). At day 180, a mean ± SD of 57.2 ± 20.9 ranging from 33.9 to 85.7 (median 57.5) was documented for the ECCO_2_R group (n=5) and of 55.0 ± 18.2 ranging from 29.6 to 78.1 (median 53.0) in the IMV group (n=5).

Table Course of SRI

| **Subscale** | **ECCO_2_R (N=8)** | **IMV (N=8)** | **Total (N=16)** |
| --- | --- | --- | --- |
| **Respiratory complaints** |  |  |  |
| *Day 60 (FU1)* |  |  |  |
| n | 8 | 7 | 15 |
| Mean ± SD | 73.0 ± 20.3 | 56.4± 24.3 | 65.3± 23.1 |
| Median (Min; Max) | 76.6 (40.6; 93.8) | 50.0 (21.9; 85.7) | 75.0 (21.9; 93.8) |
| *Day 180 (FU2)* |  |  |  |
| n | 5 | 5 | 10 |
| Mean ± SD | 61.3 ± 25.8 | 60.0 ± 13.5 | 60.6 ± 19.4 |
| Median (Min; Max) | 68.8 (31.3; 90.6) | 62.5 (40.6; 75.0) | 65.6 (31.3; 90.6) |
| **Physical functioning** |  |  |  |
| *Day 60 (FU1)* |  |  |  |
| n | 8 | 7 | 15 |
| Mean ± SD | 64.6 ± 23.3 | 38.7 ± 33.2 | 52.5 ± 30.4 |
| Median (Min; Max) | 66.7 (25.0; 91.7) | 29.2 (0.0; 79.2) | 62.5 (0.0; 91.7) |
| *Day 180 (FU2)* |  |  |  |
| n | 5 | 5 | 10 |
| Mean ± SD | 51.7 ± 29.6 | 42.5 ± 26.1 | 47.1 ± 26.7 |
| Median (Min; Max) | 58.3 (16.7; 79.2) | 33.3 (25.0; 87.5) | 37.5 (16.7; 87.5) |
| **Attendant symptoms and sleep** |  |  |  |
| *Day 60 (FU1)* |  |  |  |
| n | 8 | 7 | 15 |
| Mean ± SD | 69.2 ± 17.7 | 69.9 ± 20.4 | 69.5 ± 18.3 |
| Median (Min; Max) | 71.4 (32.1; 89.3) | 67.9 (39.3; 92.9) | 67.9 (32.1; 92.9) |
| *Day 180 (FU2)* |  |  |  |
| n | 5 | 5 | 10 |
| Mean ± SD | 61.4 ± 21.0 | 54.3 ± 32.1 | 57.9 ± 25.9 |
| Median (Min; Max) | 67.9 (28.6; 78.6) | 71.4 (14.3; 82.1) | 69.6 (14.3; 82.1) |
| **Social relationships** |  |  |  |
| *Day 60 (FU1)* |  |  |  |
| n | 8 | 7 | 15 |
| Mean ± SD | 82.8 ± 18.2 | 86.9 ± 17.3 | 84.7 ± 17.2 |
| Median (Min; Max) | 87.5 (41.7; 100.0) | 100.0 (58.3; 100.0) | 87.5 (41.7; 100.0) |
| *Day 180 (FU2)* |  |  |  |
| n | 5 | 5 | 10 |
| Mean ± SD | 71.7 ± 20.7 | 67.5 ± 16.5 | 69.6 ± 17.8 |
| Median (Min; Max) | 75.0 (37.5; 91.7) | 75.0 (45.8; 83.3) | 75.0 (37.5; 91.7) |
| **Anxiety** |  |  |  |
| *Day 60 (FU1)* |  |  |  |
| n | 8 | 7 | 15 |
| Mean ± SD | 68.1 ± 22.5 | 42.6 ± 30.2 | 56.2 ± 28.6 |
| Median (Min; Max) | 67.5 (40.0; 100.0) | 45.0 (8.3; 90.0) | 55.0 (8.3; 100.0) |
| *Day 180 (FU2)* |  |  |  |
| n | 5 | 5 | 10 |
| Mean ± SD | 32.0 ± 32.5 | 56.0 ± 34.5 | 44.0 ± 34.1 |
| Median (Min; Max) | 20.0 (5.0; 80.0) | 50.0 (10.0; 95.0) | 45.0 (5.0; 95.0) |
| **Psychological well-being** |  |  |  |
| *Day 60 (FU1)* |  |  |  |
| n | 8 | 7 | 15 |
| Mean ± SD | 71.9 ± 24.8 | 67.5 ± 14.5 | 69.8 ± 20.1 |
| Median (Min; Max) | 75.0 (36.1; 100.0) | 69.4 (47.2; 91.7) | 69.4 (36.1; 100.0) |
| *Day 180 (FU2)* |  |  |  |
| n | 5 | 5 | 10 |
| Mean ± SD | 62.2 ± 21.5 | 57.2 ± 19.9 | 59.7 ± 19.7 |
| Median (Min; Max) | 69.4 (33.3; 86.1) | 58.3 (33.3; 80.6) | 63.9 (33.3; 86.1) |
| **Social functioning** |  |  |  |
| *Day 60 (FU1)* |  |  |  |
| n | 8 | 7 | 15 |
| Mean ± SD | 71.4 ± 20.5 | 60.2 ± 24.2 | 66.2 ± 22.2 |
| Median (Min; Max) | 71.4 (46.9; 100.0) | 71.4 (28.1; 84.4) | 71.4 (28.1; 100.0) |
| *Day 180 (FU2)* |  |  |  |
| n | 5 | 5 | 10 |
| Mean ± SD | 60.0 ± 23.5 | 47.3 ± 18.2 | 53.7 ± 20.9 |
| Median (Min; Max) | 65.6 (37.5; 93.8) | 50.0 (18.8; 65.6) | 54.7 (18.8; 93.8) |
| **Summary scale** |  |  |  |
| *Day 60 (FU1)* |  |  |  |
| n | 8 | 7 | 15 |
| Mean ± SD | 71.6 ± 15.8 | 60.3 ± 19.3 | 66.3 ± 17.8 |
| Median (Min; Max) | 73.2 (46.8; 93.2) | 66.0 (35.1; 88.9) | 69.8 (35.1; 93.2) |
| *Day 180 (FU2)* |  |  |  |
| n | 5 | 5 | 10 |
| Mean ± SD | 57.2 ± 20.9 | 55.0 ± 18.2 | 56.1 ± 18.5 |
| Median (Min; Max) | 57.5 (33.9; 85.7) | 53.0 (29.6; 78.1) | 55.2 (29.6; 85.7) |

*ECCO_2_R = Extracorporeal carbon dioxide removal, FAS = full analysis set, FU = follow-up, IMV = invasive mechanical ventilation, Max = maximum, Min = minimum, SD = standard deviation, SRI = Severe Respiratory Insufficiency*

*SRI ranges from 0 to 100, a higher score reflects a better HRQoL*

**Course of EQ-5D-5L**

EQ-5D-5L utility index (results reported separately from the EQ-VAS below). The utility index was calculated from the five EQ-5D-5L dimensions (mobility, self-care, usual activities, pain/discomfort, anxiety/depression) using the official EQ-5D value sets for Germany and Spain (see SAP appendix). The calculated index at day 60 was 0.596 ± 0.389 (range 0.150–0.868; median 0.770) in the ECCO2R group (n=3) and 0.414 ± 0.451 (range 0.148–0.934; median 0.159) in the IMV group (n=3). At day 180 the mean index was 0.353 ± 0.452 in the ECCO2R group (n=2) and 0.749 ± 0.118 in the IMV group (n=3). Because only two to three patients per group provided index data and the between-group direction reversed between day 60 and day 180, no conclusion about a group difference in the utility index can be drawn.

EQ visual analogue scale (EQ-VAS, reported separately from the utility index above). The EQ-VAS was self-rated by the patients. At day 60 the mean EQ-VAS was 66.3 ± 14.1 (range 50.0–80.0; median 70.0) in the ECCO2R group and 55.0 ± 21.8 (range 20.0–85.0; median 50.0) in the IMV group. At day 180 the EQ-VAS was comparable between groups, having decreased in the ECCO2R group and increased in the IMV group. Given the small numbers, these EQ-VAS values are descriptive and do not establish that either group had a better quality of life.

Table EQ-5D-5L

| **EQ-VAS** | **ECCO_2_R (N=8)** | **IMV (N=8)** | **Total (N=16)** |
| --- | --- | --- | --- |
| *Day 60 (FU1)* |  |  |  |
| n | 8 | 7 | 15 |
| Mean ± SD | 66.3 ± 14.1 | 55.0 ± 21.8 | 61.0 ± 18.3 |
| Median (Min; Max) | 70.0 (50.0; 80.0) | 50.0 (20.0; 85.0) | 70.0 (20.0; 85.0) |
| *Day 180 (FU2)* |  |  |  |
| n | 5 | 5 | 10 |
| Mean ± SD | 60.0 ± 11.7 | 62.0 ± 13.0 | 61.0 ± 11.7 |
| Median (Min; Max) | 55.0 (50.0; 75.0) | 60.0 (50.0; 80.0) | 57.5 (50.0; 80.0) |

*ECCO_2_R = Extracorporeal carbon dioxide removal, EQ-5D-5L = 5-level EuroQol 5-dimensional questionnaire, FAS = full analysis set, FU = follow-up, IMV = invasive mechanical ventilation, Max = maximum, Min = minimum, SD = standard deviation, VAS = visual analogue scale*

*The EQ-5D-5L score ranges from 0 (worst health) to 100 (best health)*
